# Pott's disease complicated by a large retroperitoneal tuberculous mass: A case report

**DOI:** 10.1016/j.ijscr.2024.110763

**Published:** 2025-01-05

**Authors:** Salim Ouskri, Youssef Zaoui, Imad Boualaoui, Ahmed Ibrahimi, Hachem El Sayegh, Yassine Nouini

**Affiliations:** Ibn Sina University Hospital Center, Rabat, Morocco

## Abstract

**Introduction and importance:**

Tuberculosis (TB) remains a significant public health issue, especially in developing countries where its incidence is rising due to factors like overcrowding and immunosuppression. Among extrapulmonary TB forms, abdominal TB is common, while retroperitoneal TB is rare and often challenging to diagnose due to its similarity to other retroperitoneal tumors. Diagnosis typically requires invasive procedures such as laparoscopy or laparotomy.

**Case presentation:**

We report the case of a 45-year-old woman with Pott's disease leading to a large retroperitoneal tuberculous abscess. She presented with a right flank mass, chronic low back pain, weight loss, and fever. Imaging revealed spondylodiscitis at the L1-L2 vertebrae and a retroperitoneal collection. PCR confirmed *Mycobacterium tuberculosis*. Treatment involved abscess drainage and a 9-month anti-TB regimen.

**Clinical discussion:**

Retroperitoneal TB presents with non-specific symptoms, often delaying diagnosis. Imaging plays a crucial role in identifying abscesses, with CT and MRI being key tools. The treatment of retroperitoneal tuberculous abscesses includes surgical drainage and prolonged anti-tuberculosis therapy. Early diagnosis and a multidisciplinary approach are essential to managing this severe form of extrapulmonary TB.

**Conclusion:**

Retroperitoneal tuberculous abscesses, though rare, represent a severe form of extrapulmonary tuberculosis that requires increased clinical vigilance.

## Highlights

• Rising TB incidence: Tuberculosis remains a major global health issue, especially in developing countries. Abdominal TB represents 5-10% of cases, with retroperitoneal TB being rare and diagnostically challenging.
• Case details: A 45-year-old woman presented with a large right flank mass, chronic back pain, weight loss, and fever. CT and MRI revealed Pott's disease and a retroperitoneal abscess.
• Diagnosis: Diagnostic confirmation was achieved through needle aspiration and PCR, revealing *Mycobacterium tuberculosis* DNA.
• Treatment: The patient underwent abscess drainage and a 9-month course of anti-tuberculosis drugs.
• Challenges and importance of imaging: Retroperitoneal TB presents non-specific symptoms, often delaying diagnosis. CT and MRI are crucial for identifying abscesses, and PCR enhances diagnostic sensitivity.

## 1 Introduction

Tuberculosis (TB), though a curable infectious disease, remains a major public health concern, particularly in developing countries where its incidence is rising due to overcrowding, immunosuppression, and unfavorable socioeconomic factors. Among its extrapulmonary forms, abdominal tuberculosis is common, accounting for about 5–10 % of reported cases. However, retroperitoneal TB is rarely reported in the literature, posing a significant diagnostic challenge in differentiating it from other retroperitoneal tumors.

Diagnosis often requires invasive procedures such as laparoscopy or laparotomy to confirm the disease bacteriologically and histologically, enabling the initiation of anti-tuberculosis treatment [2,3].

We present a case of Pott's disease leading to a large retroperitoneal tuberculous abscess.

## 2 Case presentation

We report the case of a 45-year-old female patient, mother of two, living in a rural area, with no significant medical history, including no immunosuppression, tuberculosis, or known contact with a tuberculosis case. She had received BCG vaccination. An HIV screening test was conducted and returned negative.

The patient presented with a progressively enlarging right flank mass over 12 months, associated with chronic low back pain, a weight loss of 10 kg, and intermittent nocturnal fever.

**Fig. 1:**
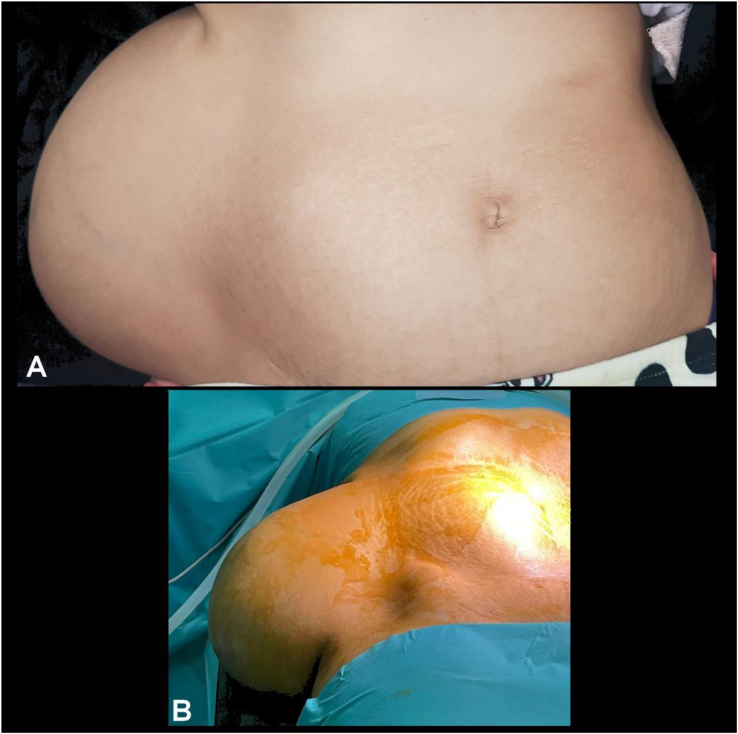
A - Large abdominal mass distorting the contour of the abdomen. B - Preoperative view.

Clinical examination revealed a patient in good general health, afebrile, slightly pale, with a large, non-tender, mobile, non-inflammatory mass in the right flank (Fig. 1). There were no lymphadenopathies or neurological deficits such as lower limb weakness, sensory levels, radiculopathies, or cauda equina syndrome. The abdomen was soft, and tenderness was noted over the lumbar vertebrae (L1,L2,L3).

Laboratory tests showed an inflammatory syndrome with a C-reactive protein (CRP) level of 70 mg/L, a white blood cell count of 12,000/μL, and hemoglobin at 10 g/dL.

**Fig. 2:**
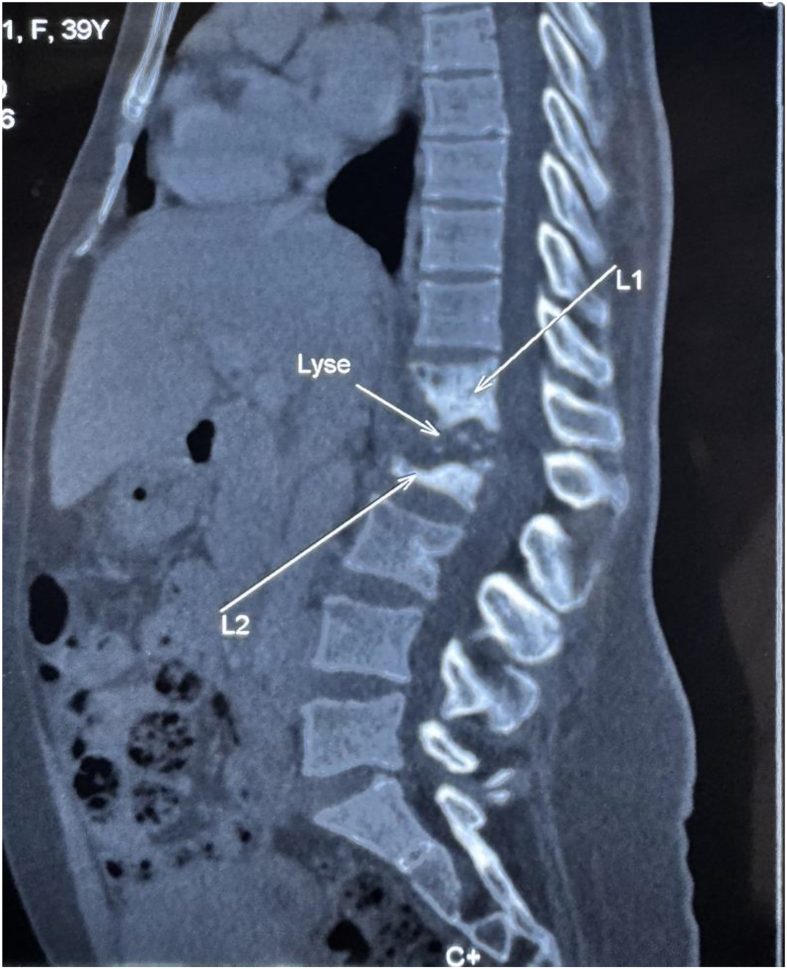
Pott's disease with lysis of both L1-L2 vertebral bodies (arrows).

**Fig. 3:**
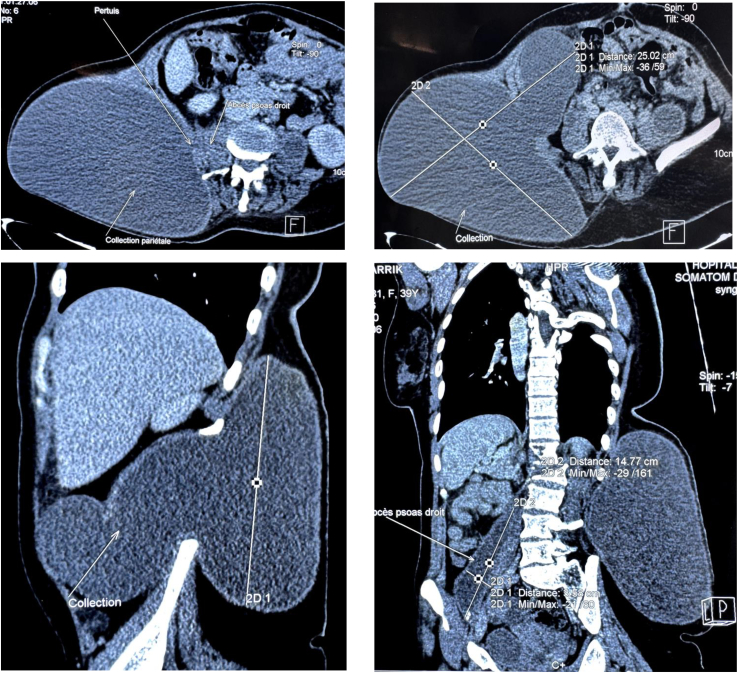
Images showing bilateral psoas abscesses and a very large retroperitoneal collection.

Abdominal computed tomography (CT) revealed spondylodiscitis involving the L1-L2 vertebrae (Fig. 2), along with bilateral psoas abscesses and multiple retroperitoneal collections, the largest measuring 180 mm × 150 mm, suggestive of Pott's disease. There were no signs of ureteral obstruction. (Fig. 3).

Spinal MRI confirmed these findings, showing a vertebral disc lesion affecting the lower plate of the first lumbar vertebra and the upper plate of the second, with lumbar kyphosis, bone lysis, and osteosclerosis. Epidural extension was also observed, with a collection exerting mass effect on the dural sac.

**Fig. 4:**
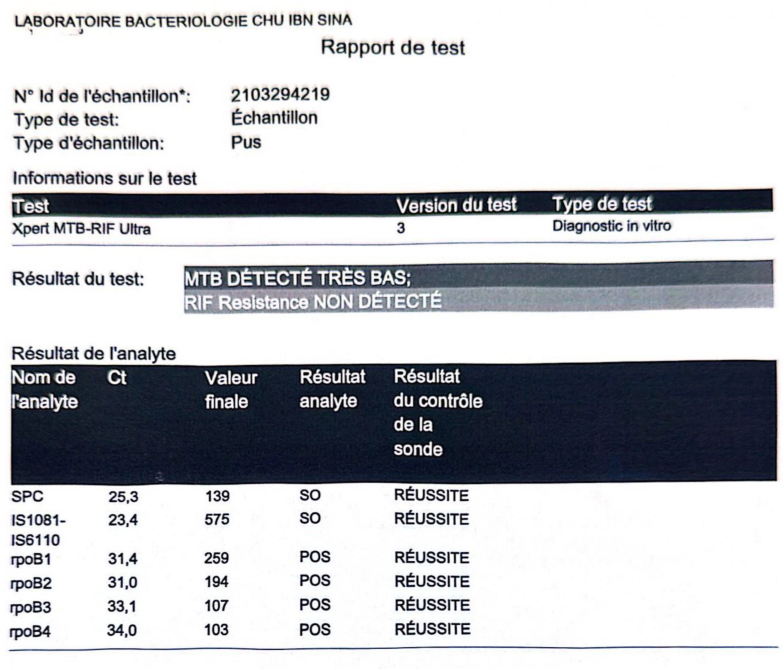
Detection of *Mycobacterium tuberculosis* circulating DNA with no evidence of the rifampicin resistance gene.

Needle aspiration of the retroperitoneal fluid revealed yellow citrine fluid. Real-time PCR analysis confirmed the presence of *Mycobacterium tuberculosis* DNA (Fig. 4). Histological analysis of the abscess wall was consistent with tissue inflammation. Chest CT showed no signs of active pulmonary tuberculosis.

Surgery was performed under general anesthesia via a retroperitoneal lombotomy, involving sectioning of the broad muscles to access the retroperitoneal space. The abscess was drained, followed by abdominal reconstruction. No surgical intervention was performed on the spondylodiscitis, as it was deemed stable by neurosurgeons.

The patient was started on anti-tuberculosis therapy consisting of rifampin, isoniazid, ethambutol, and pyrazinamide for a duration of nine months. No hematological or hepatic side effects were observed during the one-year follow-up.

The outcome was marked by significant clinical and biological improvement, with regression of inflammatory markers and improvement in general health.

## 3 Discussion

The incidence of abdominal tuberculosis has increased, particularly in HIV-positive individuals. About one-third of tuberculosis cases in Morocco are extrapulmonary, with a threefold higher frequency in HIV-positive patients [2,4,5]. The most common abdominal sites include the small intestine (44 %), cecum (35 %), and ileocecal junction (16 %) [6]. Retroperitoneal involvement is less frequent but may lead to severe complications such as urinary obstruction or spinal cord compression [7].

Patients with retroperitoneal tuberculosis may present with varied and often non-specific symptoms, making clinical diagnosis challenging. Common symptoms include abdominal pain, weight loss, low-grade fever, bloating, and urinary symptoms such as hematuria and low back pain [4,15]. In advanced cases, nerve compression may result in lower limb paralysis, as reported in some studies [4,6].

Diagnosis of retroperitoneal tuberculosis relies on a combination of clinical, radiological, and histopathological criteria. However, due to the non-specific nature of symptoms, diagnosis is often delayed.

Clinical manifestations such as anemia and inflammatory syndrome are non-specific and do not confirm the diagnosis [15]. The tuberculin skin test may suggest TB infection but is insufficient to confirm retroperitoneal involvement [16].

Imaging plays a crucial role in detecting retroperitoneal tuberculous abscesses. CT and abdominal ultrasound are the primary modalities used. CT can reveal bowel wall thickening, retroperitoneal lymphadenopathy, or pseudo-tumoral masses [16,18]. MRI, though non-specific, may show hypointense T1 lesions with variable T2 signals, aiding in assessing fibrosis extent [16].

More invasive procedures such as laparoscopy, or if not possible, laparotomy, are essential for biopsy to identify characteristic caseating granulomas and for culture to isolate *Mycobacterium tuberculosis* [[19], [20], [21]]. PCR testing on biopsy samples has improved diagnostic sensitivity, reaching 75–80 %, with high specificity of 85–95 % [21].

The treatment of retroperitoneal tuberculous abscesses involves surgical drainage or aspiration, along with prolonged anti-tuberculosis polytherapy, aiming to eradicate the Koch bacillus, particularly in cases with complications such as ureteral obstruction or renal failure [6,9].

## 4 Conclusion

Retroperitoneal tuberculous abscesses, though rare, represent a severe form of extrapulmonary tuberculosis that requires increased clinical vigilance. The diversity of clinical presentations and their resemblance to other pathologies make early diagnosis particularly difficult. A multidisciplinary diagnostic approach, integrating advanced imaging techniques, and histological analyses, is essential for accurate diagnosis and appropriate treatment initiation. Long-term follow-up is crucial to monitor disease progression and prevent irreversible complications.

## CRediT authorship contribution statement

Salim Ouskri Urology Resident: FIRST author has contributed in the writing and correction the case report

Youssef Zaoui Urology Resident: has contributed in the correction the case report

Imad Boualaoui Urology assistant Professor / has contributed in the correction the case report

Ahmed IBRAHIMI Urology assistant Professor: SECOND author has contributed in the writing and correction the case report

Hachem El Sayegh Urology Professor: has contributed in the correction the case report

Yassine Nouini Urology Professor: has contributed in the correction the case report

## Ethical approval

Ethical approval for this study was provided by the Ethical Committee of **IBN SINA** University Hospitals, Rabat, Morocco on 10/10/2024.

## Guarantor

Salim Ouskri

## Funding

NA.

## Methods

This the work has been reported in line with the SCARE criteria.

## Declaration of competing interest

NA.
